# Supplementary material for: Highly accurate retinotopic maps of the physiological blind spot in human visual cortex
Source: Hum Brain Mapp. 2022 Jul 7;43(17):5111–25. doi: 10.1002/hbm.25996 (PMC9812231; doi:10.1002/hbm.25996)
Supplement: Supplementary file 1 — Appendix S1 Figure S1 Distributions of goodness‐of‐fit values (smoothed with a kernel density estimator) for all analyzed voxels. (a) Peak correlation in reverse correlation profiles (R 2 RC). (b). Goodness‐of‐fit for 2D pRF models fit to reverse correlation profiles (R 2 pRF). (c) Goodness‐of‐fit, normalized relative to noise ceiling (nR 2) in the forward‐modelling analysis. Individual curves denote distributions from individual participants. Color code denotes condition (control, blind spot) and field strength (3T, 7T). The dashed vertical black line indicates the statistical threshold applied to each measure Figure S2 Reconstruction of retinotopic maps in visual space based on forward modelling results. For each supra‐threshold pRF, we generated the pRF profile from its parameter estimates. The heat map indicates the density of pRF profiles at a given visual field location. Coordinates are in degrees of visual angle relative to the blind spot center. Data from four participants are shown in columns. (a) Control eye stimulation. (b) Blind spot stimulation. Green dots denote the outline of the blind spot as determined by the behavioral localizer. Note that unlike in the reverse correlation analysis, it is possible to fit pRF centers outside the stimulated part of the visual field, or even outside the aperture space. Therefore, these reconstructions are not confined to the circular region containing the pRF stimulus Table S1 Behavioural blind spot localization for each participant. All values are in degrees visual angle. Fixation denotes the side of the screen participants were instructed to fixate. X and Y denote the horizontal and vertical visual field location of the blind spot centroid, respectively. Asterisks next to participant IDs indicate participants scanned at 7T [file HBM-43-5111-s001.docx]

**Supplementary Information**


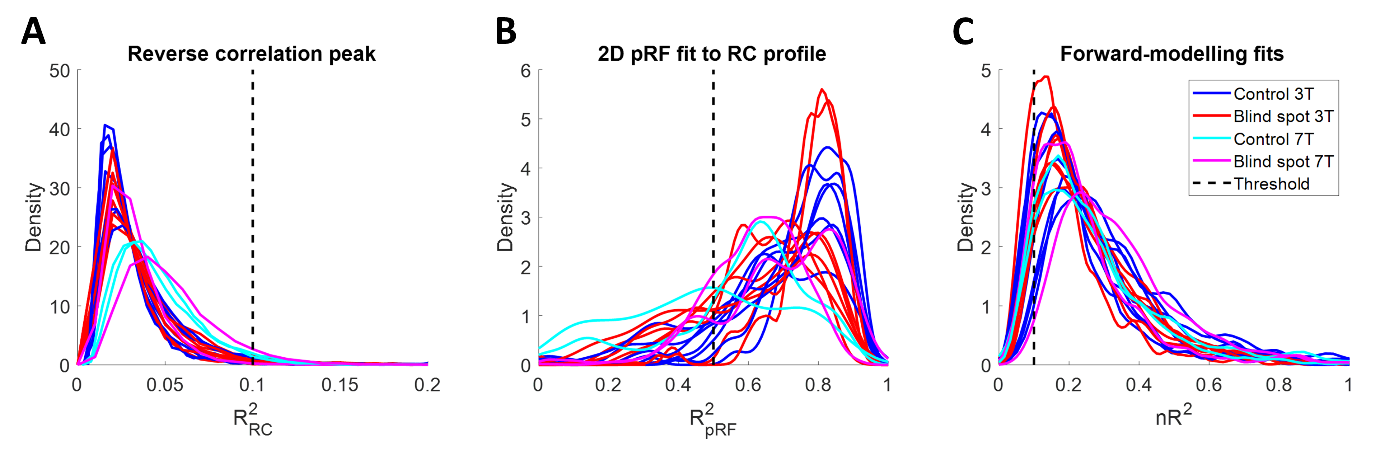


**Supplementary Figure S1.** Distributions of goodness-of-fit values (smoothed with a kernel density estimator) for all analyzed voxels. A. Peak correlation in reverse correlation profiles (R^2^_RC_). B. Goodness-of-fit for 2D pRF models fit to reverse correlation profiles (R^2^_pRF_). C. Goodness-of-fit, normalized relative to noise ceiling (nR^2^) in the forward-modelling analysis. Individual curves denote distributions from individual participants. Color code denotes condition (control, blind spot) and field strength (3T, 7T). The dashed vertical black line indicates the statistical threshold applied to each measure.


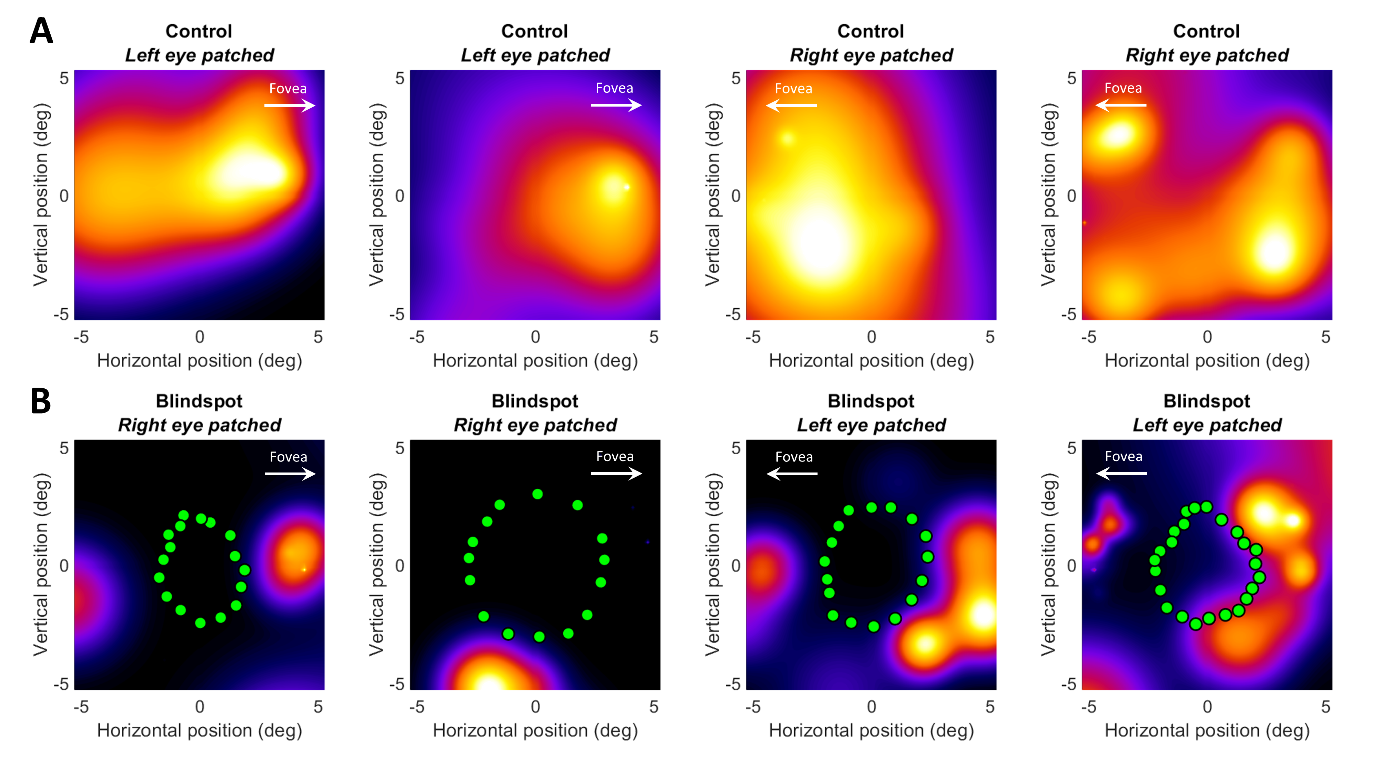


**Supplementary Figure S2.** Reconstruction of retinotopic maps in visual space based on forward modelling results. For each supra-threshold pRF, we generated the pRF profile from its parameter estimates. The heat map indicates the density of pRF profiles at a given visual field location. Coordinates are in degrees of visual angle relative to the blind spot center. Data from four participants are shown in columns. **A.** Control eye stimulation. **B.** Blind spot stimulation. Green dots denote the outline of the blind spot as determined by the behavioral localizer. Note that unlike in the reverse correlation analysis, it is possible to fit pRF centers outside the stimulated part of the visual field, or even outside the aperture space. Therefore, these reconstructions are not confined to the circular region containing the pRF stimulus.

**Supplementary Table S1.** Behavioural blind spot localization for each participant. All values are in degrees visual angle. *Fixation* denotes the side of the screen participants were instructed to fixate. *X* and *Y* denote the horizontal and vertical visual field location of the blind spot centroid, respectively. Asterisks next to participant IDs indicate participants scanned at 7T.

| **ID** | **Fixation** | **X** | **Y** | **Eccentricity** | **Area** | **Width** | **Height** |
| --- | --- | --- | --- | --- | --- | --- | --- |
| *02L* | Left | 16.1 | -1.0 | 16.1 | 20.5 | 4.7 | 5.8 |
| *04R* | Right | -14.4 | -2.0 | 14.6 | 31.8 | 5.9 | 7.0 |
| *05L* | Left | 16.0 | -1.7 | 16.1 | 20.9 | 5.2 | 5.8 |
| *06R* | Right | -16.6 | -1.4 | 16.7 | 37.0 | 6.7 | 7.0 |
| *97L* | Left | 14.4 | -1.3 | 14.4 | 23.6 | 5.1 | 6.4 |
| *98L* | Left | 15.6 | 0.7 | 15.6 | 23.4 | 5.1 | 5.9 |
| *99R* | Right | -14.2 | -0.6 | 14.2 | 16.1 | 4.2 | 5.3 |
| *11L** | Left | 19.0 | 0.2 | 19.0 | 23.5 | 5.2 | 5.9 |
| *12L** | Left | 16.1 | -0.3 | 16.1 | 19.9 | 4.9 | 5.5 |
